# Pericytes contribute to the islet basement membranes to promote beta-cell gene expression

**DOI:** 10.1038/s41598-021-81774-8

**Published:** 2021-01-27

**Authors:** Lina Sakhneny, Alona Epshtein, Limor Landsman

**Affiliations:** Department of Cell and Developmental Biology, Sackler Faculty of Medicine, Tel Aviv University, 69978 Ramat Aviv, Tel Aviv, Israel

**Keywords:** Molecular biology, Physiology, Metabolism, Cell biology, Extracellular signalling molecules

## Abstract

β-Cells depend on the islet basement membrane (BM). While some islet BM components are produced by endothelial cells (ECs), the source of others remains unknown. Pancreatic pericytes directly support β-cells through mostly unidentified secreted factors. Thus, we hypothesized that pericytes regulate β-cells through the production of BM components. Here, we show that pericytes produce multiple components of the mouse pancreatic and islet interstitial and BM matrices. Several of the pericyte-produced ECM components were previously implicated in β-cell physiology, including collagen IV, laminins, proteoglycans, fibronectin, nidogen, and hyaluronan. Compared to ECs, pancreatic pericytes produce significantly higher levels of α2 and α4 laminin chains, which constitute the peri-islet and vascular BM. We further found that the pericytic laminin isoforms differentially regulate mouse β-cells. Whereas α2 laminins promoted islet cell clustering, they did not affect gene expression. In contrast, culturing on Laminin-421 induced the expression of β-cell genes, including *Ins1*, *MafA*, and *Glut2*, and significantly improved glucose-stimulated insulin secretion. Thus, alongside ECs, pericytes are a significant source of the islet BM, which is essential for proper β-cell function.

## Introduction

β-Cells depend on the islet niche for their mass and functionality. A central constituent of the islet niche is the extracellular matrix (ECM)^1–5^. In addition to providing biomechanical support to the islets, ECM components are required for β-cell development, proliferation, survival, and proper insulin secretion. The ECM regulates β-cell gene expression directly through binding specific receptors on these cells, and indirectly, through the storage of signaling molecules, such as growth factors^1,3,5^. Loss of the ECM during the islet isolation process is considered to be a significant factor in the poor engraftment rate of transplanted β-cells, thus limiting cell replacement therapy to diabetes^6^. Hence, recapitulating the islet ECM in both isolated islets and stem-cell-derived β-cells is of high therapeutic value. However, while many studies demonstrated the importance of the ECM, its interactions with β-cells are still not fully understood.

Two major ECM classes exist, the interstitial matrix (IM) and the basement membrane (BM)^7^. IMs are found in the space between the tissue stroma cells and contain, among other components, fibrillar collagens and fibronectin. BMs are specialized structures that underlie epithelia and surround organs and mainly comprise collagen IV, laminins, and heparan sulfate proteoglycans (HSPG). Islets have two distinct BMs: the peri-islet membrane and the vascular BM, which differ in both their anatomic location and molecular composition^3,4^. The peri-islet membrane, which encapsulates the islets, separates the endocrine cells from the exocrine pancreas and serves as a barrier from immune cell infiltration to the islets. The vascular BM surrounds the islet capillary network and directly contacts mouse endocrine cells. Human islets are characterized by a double-layered BM, consists of a vascular BM layer covered by an invagination of the peri-islet membrane^8,9^. Thus, human endocrine cells are not in direct contact with the vascular BM components but with the invaginated peri-islet membrane.

β-Cells directly respond to BM components through both integrin and non-integrin receptors -mediated signaling^1,2,4,5^. For example, β1 integrin, a significant component of the integrin dimer expressed by β-cells, is required for these cells' development and expansion, as well as for insulin production and secretion^2,10–13^. While integrins bind multiple ECM components, including collagens and fibronectin, their interactions with laminins are potentially more significant^4^. The interactions with the various BM components differentially affect β-cells, although the underlying mechanism is largely unknown.

The specific expression and distribution of the different BM components in the islets were yet to be fully characterized. It has been shown, however, that β-cells do not produce BM components^1,2^. The islet BM composition displays temporal and spatial patterns. For example, HSPGs can be found in the adult islets, but not the embryonic or newborn pancreata^14^. On the other hand, Laminin (LM)-111 is the primary isoform expressed in the embryonic pancreas but is absent from the adult tissue^4,8,15^. Laminins are heterotrimers composed of α, β, and γ chains, when the α chain largely dictates receptor specificity^16^. Three different types of α (α2, α4, and α5), two of β (β1 and β2), and one of γ (γ1) laminin chains were detected in the adult islets, potentially forming six different isoforms^8^. The various laminin isoforms in the islets are found at different locations. In humans, the islet vascular BM comprises α4 and α5 laminin chains, while the peri-islet membrane contains α2 and α5 laminin chains^8^. In the mouse, α2 laminins can also be found in the vascular BM^17,18^. Similarly, the β1 and γ1 laminin chains comprise both the peri-islet membrane and vascular BM, whereas the β2 chain is specific to the latter^8,17,18^. The additional key component of the islet ECM, collagen IV, constitutes both the peri-islet and vascular BM^2,5^. Which cells produce the various islet BM components is still unclear.

Endothelial cells (ECs) were long known to contribute to the islet BM. Pancreatic ECs produce some components of the islet BM, including collagen IV and the α4 and α5 laminin chains, to regulate insulin production^1,2^. However, an additional source of the islet BM has been suggested^9^. Pericytes are found within and around islets, where they are associated with ECs. Pancreatic pericytes were shown by others and us to control insulin secretion, both indirectly through the modulation of the islet blood flow and directly, through the production of signaling molecules that regulate β-cell gene expression and function^19–22^. The identity of the pericyte-produced β-cell regulatory factors is only now begun to unveil. While pericytes are considered to contribute to the vascular BM throughout the body, their level of contribution is unclear^16^. Furthermore, whether pancreatic pericytes are capable of producing components of the vascular and peri-islet BM is unknown.

Here, we set to determine the contribution of pericytes to the islet ECM. First, we utilized RNAseq analysis to profile their expression of ECM components. Our analysis pointed to the production of multiple constituents of both pancreatic IMs and BMs by pericytes. We then focused on the main components of the islet BM, collagen IV and laminins. While both ECs and pericytes produce collagen IV, we show that pericytes are the primary source of two of the three pancreatic laminin α chains: α2 and α4. To determine the effect of these laminins, we cultured mouse islet cells in their presence. Pericytic laminins, in particular α2 laminins, promoted islet cell clustering. The α4 laminin LM-421, but not other tested laminin isoforms, induced the expression of β-cell genes and stimulated glucose-stimulated insulin secretion. Thus, our analysis indicated a significant contribution of pericytes to the pancreatic and islet ECM and pointed to the differential role of pericytic laminins in β-cell organization and function.

## Results

### Pancreatic pericytes express components of the interstitial matrix

**Figure 1:**
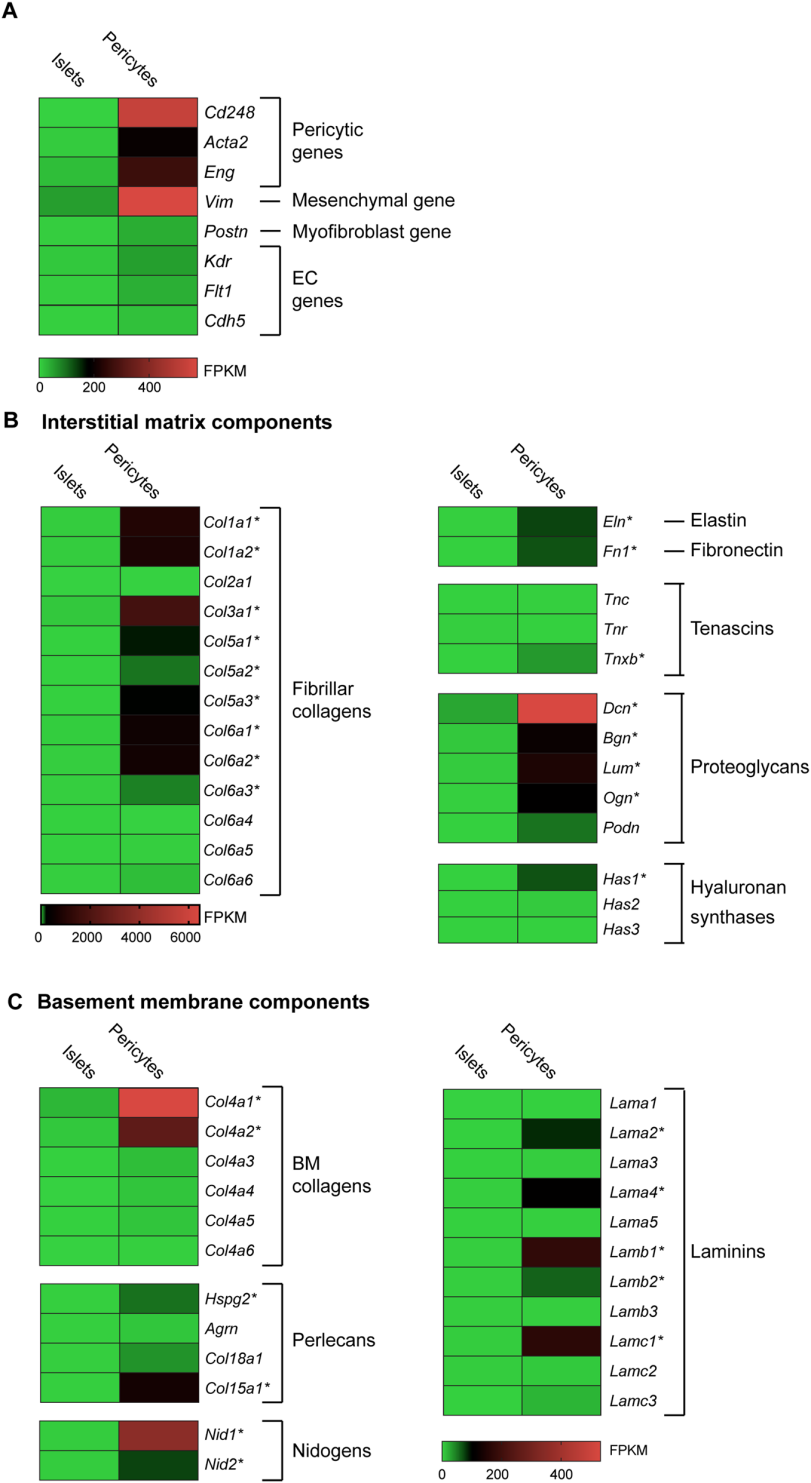
Pancreatic pericytes express interstitial matrix and basement membrane components. RNAseq analysis of pericytes (FACS-purified from the pancreatic tissues of *Nkx3.2*-Cre;*R26*-YFP mice based on their YFP labeling) and islets (isolated from wild-type mice), as previously described^21^. Heat maps show mean expression levels (as fragments per kilobase of exon per million aligned fragments [FPKM]) of indicated genes (N = 3; each represents cells of a single mouse). *, Genes exhibiting average pericytic expression levels higher than 50 FPKM and statistically significant differences between pancreatic pericytes and islets.

In IMs, proteins as collagens, fibronectin, elastin, and tenascin determine the characteristic fibrous networks, while proteoglycans dictate interstitial spaces^7^. To assess pericytes' contribution to the pancreatic IM, we profiled their gene expression employing an RNAseq analysis (previously described in Ref.^21^). In this array, we analyzed pancreatic mural cells, labeled by Nkx3.2/YFP, which primarily constitute pericytes (~ 85%) in addition to the closely related vascular Smooth Muscle cells (vSMCs)^20,21,23^. Islets, which contain primarily pancreatic endocrine cells, served as controls. Indeed, pericytic genes, including *Cd248*, *Acta2,* and *Eng* (encoding Endosialin, Smooth Muscle Actin α2, and Endoglin, respectively)^24^, were enriched in the pericyte population (Fig. 1A). Corresponding with pericytes being a primary mesenchymal population in the pancreas^21,25,26^, these cells express *Vim* (encoding Vimentin; Fig. 1A). Pericytes can convert to fibroblasts, including pancreatic myofibroblasts, under pathophysiological conditions^27,28^. Analyzed pericytes were isolated from naïve animals, and indeed the expression of *Postn*, encoding the myofibroblast marker Periostin^28^, is low in these cells (Fig. 1A). ECs are not labeled by Nkx3.2/YFP and can be separated from cells that do by flowcytometry^20,29^. Indeed, the expression of the EC genes *Kdr*, *Flt1*, and *Cdh5* (encoding VEGFR 1 and 2, and VE-cadherin, respectively) was low in the analyzed pericytic population (Fig. 1A).

To evaluate the contribution of pericytes to the pancreatic IM, we focused on genes encoding its various components^7^. Our analysis revealed the pancreatic pericytes express substantial levels of genes encoding fibrillar collagens. In particular, these cells expressed genes encoding collagens I, III, V, and VI (*Col1a1, Col1a2, Col3a1, Col5a1, Col5a2, Col5a3, Col6a1, Col6a2,* and *Col6a3*, respectively) and did so in significantly higher levels than endocrine cells do (Fig. 1B). The predominant fibrillar collagen isoform expressed by pancreatic pericytes, in terms of its expression levels, was collagen III.

Pancreatic pericytes further expressed genes encoding Elastin, Fibronectin, and Tenascin-X (*Eln*, *Fn1*, and *Tnxb*, respectively) at higher levels than endocrine cells do (Fig. 1B). Genes encoding various proteoglycans that contribute to IMs, including Decorin, Biglycan, Lumican, Osteoglycin, and Podocan (*Dcn*, *Bgn*, *Lum*, *Ogn*, and *Podn*, respectively) were also expressed by pancreatic pericytes (Fig. 1B). The predominant proteoglycan that is expressed by these cells was Decorin.

Finally, pancreatic pericytes expressed hyaluronan synthase 1, encoded by *Has1*, at significantly higher levels than expressed by islet cells (Fig. 1B). Hyaluronan, also known as a Hyaluronic acid (HA), is a polysaccharide that comprises the peri-islet capsule. HA was shown to act as a barrier of leukocyte infiltrations to the islets, which is lost during T1D^30^.

To conclude, our gene expression analysis pointed to the contribution of pericytes to the various pancreatic IM components, including fibrillar collagens, fibronectin, elastin, tenascin, and proteoglycans.

### Pericytes express components of the islet basement membranes

Pancreatic pericytes are enriched with genes involved in integrin signaling^21^. BMs are specialized ECMs consisting of core proteins organized into sheet-like networks of interconnected collagen IV, laminins, perlecans, and nidogens^7^. While some of these components contribute to both the vascular BM and the peri-islet membrane, others contribute to only one of these two membranes^8,17,18^. To evaluate the contribution of pericytes to the pancreatic BMs, we profiled on the expression of genes encoding their various components, as described above.

Our RNAseq analysis indicated that pancreatic pericytes expressed genes encoding the two predominant collagen IV chains, *Col4a1* and *Col4a2*, in considerably higher levels than islet cells (Fig. 1C). Pancreatic pericytes also expressed genes encoding various perlecans, including HSPG2 (encoded by *Hspg2*) and collagen XV (encoded by *Col15a1*)(Fig. 1C). Our analysis further revealed the expression of both Nidogen 1 and 2 (encoded by *Nid1* and *Nid2*, respectively) by pancreatic pericytes at significantly higher levels than in islets (Fig. 1C).

Along with collagen IV, laminins constitute a significant BM component and are therefore abundant in both the peri-islet capsule and within the islets^4^. Our analysis revealed that pancreatic pericytes expressed two of the three known islet α laminin chains, α2 and α4 (encoded by *Lama2* and *Lama4*, respectively). Pericytes further express the two islet β chains, β1 and β2 (encoded by *Lamb1* and *Lamb2*, respectively), and the single detected γ chain, γ1 (encoded by *Lamc1*)(Fig. 1C). Thus, pericytes express all the known islet laminins, but the α5 laminin chain. Therefore, our analysis indicates that pancreatic pericytes express laminin chains that comprise four isoforms: LM -211, -221, -411, and -421 (also known as LM -2, -4, -8, and -9, respectively).

To conclude, pancreatic pericytes express multiple components of the peri-islet membrane and vascular BM, including collagen IV, laminins, perlecans, and Nidogens (Summarized in Table 1).

**Table 1:** A summary of the primary islet BM components expression patterns.

BM component	Expression by ECs	Expression by Pericytes	Reference
Collagenase IV	+ +	+	Ref.^2^; Figs. 1, 2
Laminin, α1	–	–	Ref.^2^; Fig. 1
Laminin, α2	–	+ +	Ref.^2^; Figs. 1, 2
Laminin, α3	–	–	Ref.^2^; Fig. 1
Laminin, α4	+	+ +	Ref.^2^; Figs. 1, 2
Laminin, α5	+	–	Ref.^2^; Fig. 1
Nidogens	?	+	Figure 1
HSPG	?	+	Figure 1

### The laminin α2 chain co-localizes with islet pericytes

**Figure 2:**
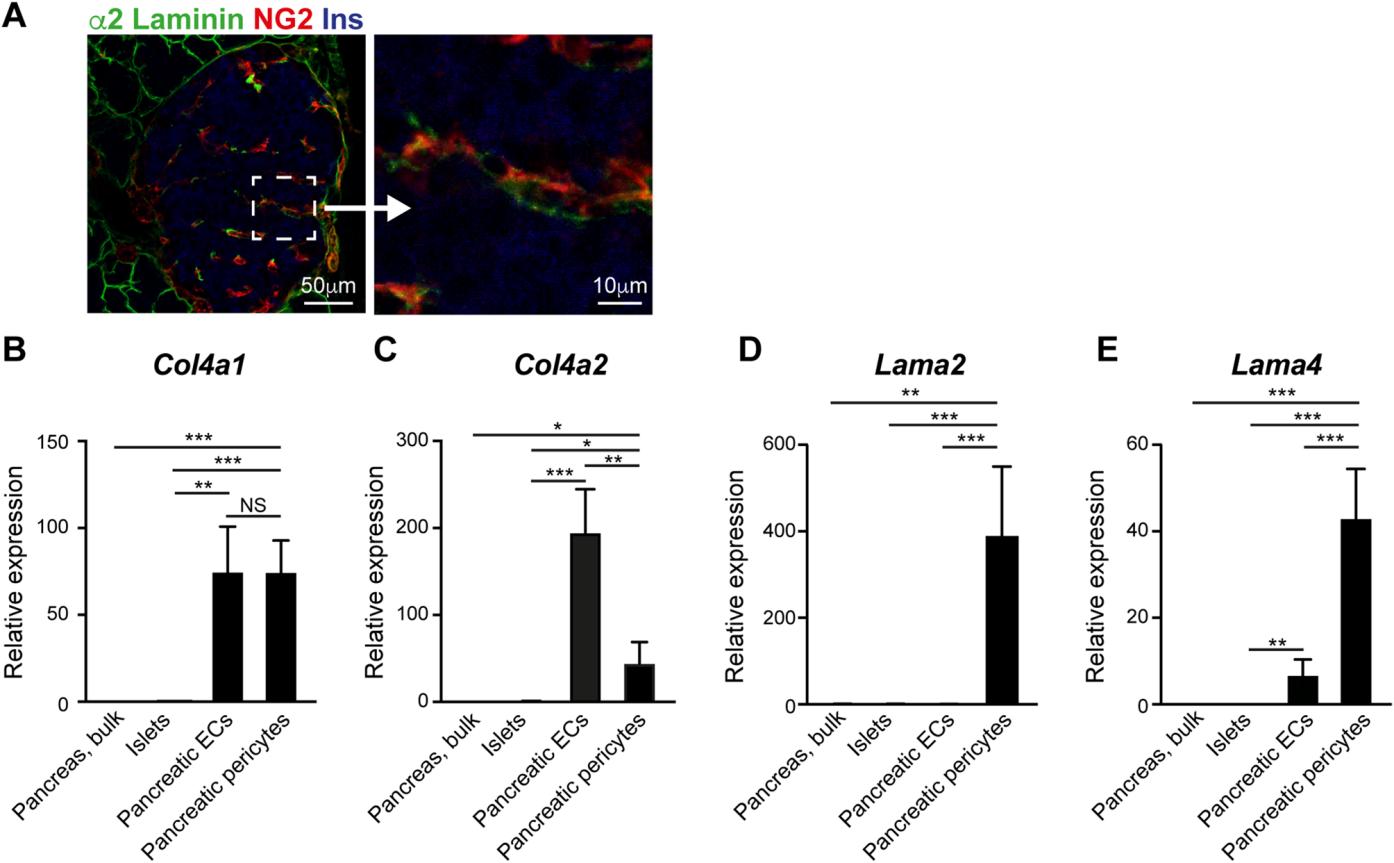
Pancreatic pericytes and endothelial cells differ in their expression of BM components. (**A**) Immunofluorescence analysis of mouse pancreatic tissue for α2 laminin (green), the pericytic marker NG2 (red), and insulin (blue). Right panel shows a higher magnification of the area demarked by a dashed line. (**B**–**E**) Bar diagrams show expression levels of indicated genes determined by qPCR. RNA was extracted from bulk pancreatic tissues, islets (average was set to '1’), pancreatic endothelial cells (ECs), and pancreatic pericytes (isolated as described in Fig. 1). N = 3–5. *, p < 0.05; **, p < 0.001; ***, p < 0.005.

Three different laminin α chains were detected in the adult islet: α2, α4, and α5^8^. While α4 and α5 laminin chains were shown to be expressed by ECs^2^, the source of the α2 chain remained unknown. Our RNAseq analysis indicated that pancreatic pericytes express this laminin chain. To validate this finding in the protein level, we stained mouse pancreatic tissue for insulin, the pericytic marker NG2, and the α2 laminin chain. As shown in Fig. 2A, the α2 laminin chain co-localized with islet pericytes, in agreement with these cells being the source of this BM component.

### Pancreatic α2 and α4 laminin chains are predominantly expressed by pericytes

The islet capillary network is comprised of both pericytes and ECs, found within and surrounding the islets^31^. ECs produce major components of the pancreatic vascular BM and peri-islet membrane^1,2^. Specifically, these cells were shown to express genes encoding the α1 and α2 collagen IV chains and the α4 and α5 laminin chains^2^. Thus, to assess the relative contribution of pericytes to the pancreatic BMs, we directly compared their expression of selected genes to pancreatic ECs by qPCR. As a control, gene expression was also analyzed in isolated islets, which primarily contain endocrine cells, and bulk pancreatic tissue, which primarily contains exocrine cells.

First, we verified the pericytic expression of *Col41a1*, *Col4a2*, *Lama2*, and *Lama4*. Similar to the RNAseq analysis, our qPCR analysis pointed to significantly higher expression levels of all four analyzed genes in pancreatic pericytes than isolated islets and bulk pancreatic tissue (Fig. 2). Of note, the pericytic expression levels of *Lama2* were two orders of magnitude higher than of islets (390-fold; Fig. 2D) and that of *Lama4, Col4a1,* and *Col4a2* one order of magnitude higher (44-, 74-, and 30-fold, respectively; Fig. 2B,C,E). Thus, pericytes are a significant pancreatic source of laminins and collagen IV.

Next, we directly compared the expression of selected genes in pancreatic pericytes and ECs. In agreement with current literature, pancreatic ECs expressed *Lama4*, *Col4a1*, and *Col4a2*, but not *Lama2*, and do so at significantly higher levels than islet cells (Fig. 2D)^2^. The two analyzed collagen IV chains were expressed by both pericytes and ECs (Fig. 2B,C). However, while the two cell populations displayed similar *Col4a1* transcripts levels, ECs expressed significantly higher levels (4-fold) of *Col4a2* than pericytes did. Pancreatic pericytes expressed *Lama4* at significantly higher levels than pancreatic ECs did (6-fold; Fig. 2E). To conclude, our analysis indicates that both pericytes and ECs contribute to the collagen IV found in the islet BMs. In contrast, pericytes are the predominant source of α2 and α4 Laminins in the pancreas (summarized in Table 1).

### α2 laminins stimulate islet cell clustering

Laminins containing the α5 and α1 chains were shown to promote β-cell survival and proliferation, as well as insulin production^1,2,4,32^. To research similar effects of α2 and α4 laminins directly, we cultured islet cells in the presence of these components, in the form of recombinant proteins. To minimize the influence of the islet native BM, isolated islets were dispersed into single cells. As pericytes also produce β1, β2, and γ1 laminin chains (Fig. 1C), we analyzed all laminin isoforms containing these chains, namely: LM -211, -221, -411, and -421.

**Figure 3:**
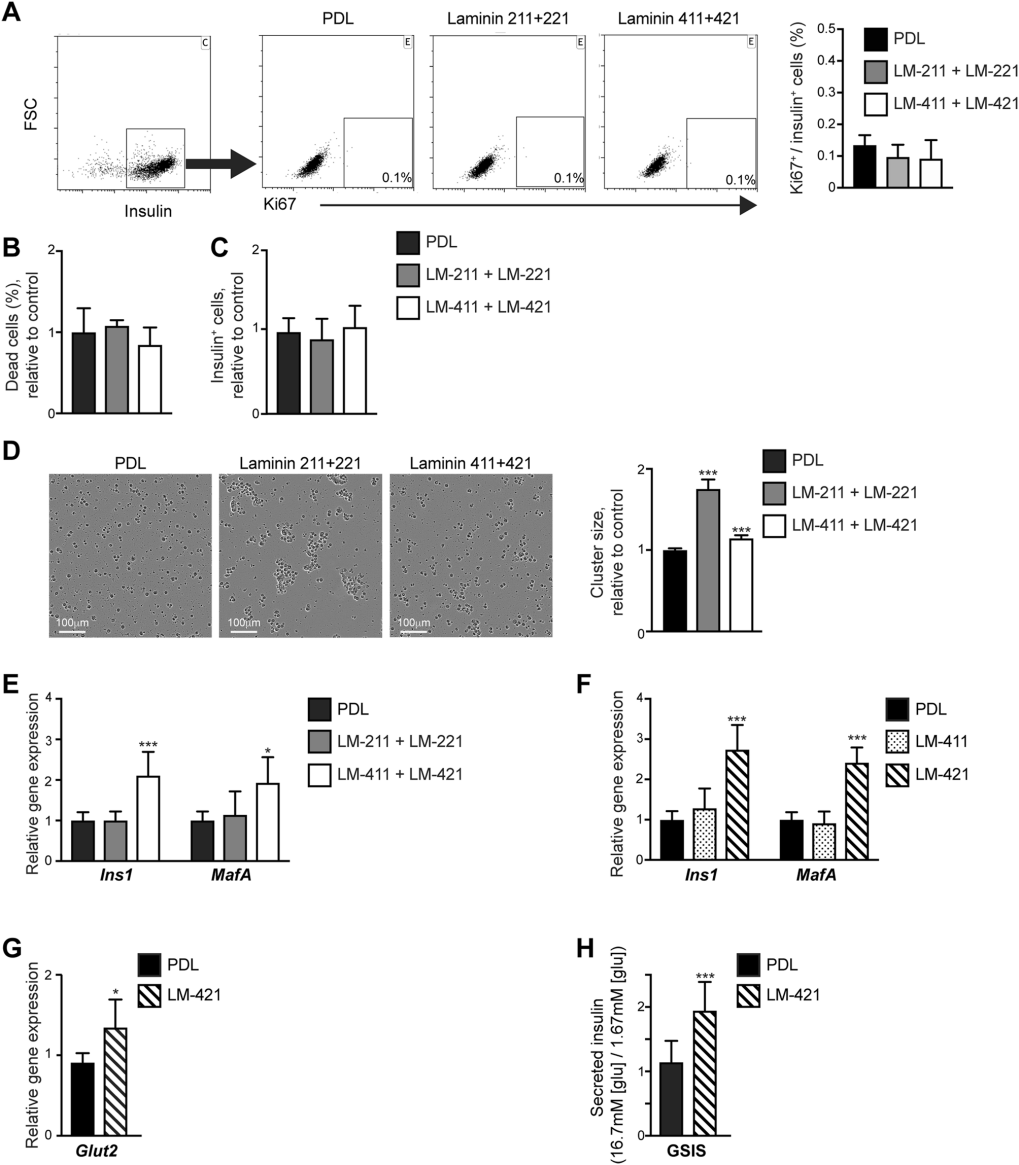
Pericytic laminins influence islet cells clustering, gene expression, and function. Dispersed mouse islet cells were cultured on either Poly-D-Lysine (PDL) or indicated recombinant laminin combinations (LM): LM-211 and -221 mixture (gray bars); LM-411 and -421 mixture (empty bars); LM-411 (dotted bars); or LM-421 (striped bars). (**A**) After cultured for 48 h, cells were stained for insulin and the proliferating cell marker Ki67 and analyzed by flow cytometry. *Left*, a representative dot plot of insulin staining. *Middle*, representative dot plots of Ki67 labeling of insulin^+^ cells under each culture condition. *Right*, a bar diagram (mean ± SD) shows the percentage of Ki67^+^ cells of insulin^+^ cells. n = 4. (**B**) Bar diagram (mean ± SD) shows the relative percentage of dead cells upon culturing on PDL (the average was set to '1’) or laminins. Dead cells were identified as DAPI^+^ unfixed cells. n = 3–4. C, Bar diagram (mean ± SD) shows the relative number of insulin-expressing cells upon culturing on PDL (the average was set to ‘1’) and laminins. n = 5. (**D**) Clustering of dispersed islet cells for 72 h. *Left*, representative images. *Right*, bar diagram (± SEM) shows the relative cluster size. n > 600 clusters for each condition. (**E**–**G**) Bar diagram shows a qPCR analysis of indicated genes upon culturing for 72 h. The averages of PDL-cultured cells were set to '1’. n = 4–5. (**H**) Bar diagram shows glucose-stimulated insulin secretion (GSIS) analysis. After a 72-h culture on either PDL or LM-421, cells were incubated with low (1.67 mM) glucose concentration, followed by incubation with high (16.7 mM) glucose concentration. The levels of secreted insulin were measured, and the ratio between levels secreted in response to high and low glucose levels was determined for each well. n = 6–7. *, p < 0.05; ***, p < 0.005, as compared to PDL-treated cells.

Dispersed mouse islet cells were seeded on plates coated with α2 laminins (a mixture of recombinant LM-211 and LM-221), α4 laminins (a mixture of recombinant LM-411 and LM-421), or control PDL. To determine potential changes in β-cell proliferation, cells were stained for insulin and the proliferating cell marker Ki67, and the percentage of proliferating β-cells was determined by flow cytometry. As shown in Fig. 3A, we could not detect cell proliferation after 48 h of culturing on analyzed laminins. Cell death was comparable on PDL and laminins coated plates (Fig. 3B). In agreement, culturing on α2 or α4 laminins did not affect the number of insulin-expressing cells in cultured islet cells (Fig. 3C).

Whereas islet cells cultured on PDL remained dispersed, these cultured on laminins clustered (Fig. 3D). Culturing on α4 laminins slightly (by 15%) increased the average cell cluster size compared to controls (Fig. 3D). However, culturing on α2 laminins more predominantly promoted clustering of islet cells, as indicated by the presence of large cell clusters and a 75% increase in the average cluster size (Fig. 3D).

To conclude, laminins naturally produced by pancreatic pericytes do not support β-cell expansion and survival but promote the clustering of islet cells, thus potentially fostering cell–cell interactions.

### LM-421 promotes β-cell gene expression and glucose-stimulated insulin secretion

Next, we aimed at testing the ability of pericytic laminins to support β-cell gene expression. To this end, we analyzed the effect of α2 and α4 laminins on the expression of insulin and MafA, a prominent transcription factor required for mature β-cell phenotype and proper function^33^. RNA was extracted from dispersed islet cells cultured on PDL, α2 (LM-211 and LM-221), and α4 Laminins (LM-411 and LM-421) for 72 h, and *Ins1* and *MafA* transcript levels were analyzed by qPCR (Fig. 3E). Our analysis shows higher expression levels of both these genes in islet cells cultured on α4 Laminins than in these cultured on PDL. In contrast, *Ins1* and *MafA* transcript levels were comparable in cells cultured on PDL and α2 Laminin (Fig. 3E).

Pericytes express both the β1 and β2 laminin chains, thus producing components of two α4 Laminins, LM-411 and LM-421 (Fig. 1C). We, therefore, set to analyze the specific effect of LM-411 and LM-421 to determine which is capable of promoting β-cell gene expression. To this end, dispersed islet cells were cultured on PDL, LM-411, and LM-421 for 72 h, and their RNA was extracted for gene expression analysis. As shown in Fig. 3F, while cells cultured on LM-411 expressed *Ins1* and *MafA* at similar levels of control cells, cells cultured on LM-421 expressed significantly higher levels of both β-cell genes. Culturing on LM-421 further improved the expression of the glucose transporter Glut2 in islet cells, which is required for proper β-cell glucose sensing (Fig. 3G). Thus, LM-421, but not LM-411, promotes the expression of β-cell genes.

Next, we set to test whether LM-421 supports the functionality of cultured β-cells. To this end, dispersed islet cells were cultured on either PDL or LM-421. After 72 h of culture, cells were exposed to low (1.67 mM) and high (16.7 mM) glucose concentrations, and the level of secreted insulin was measured and compared. As shown in Fig. 3H, cells cultured on LM-421 displayed improved glucose-stimulated insulin secretion capabilities compared to control. Thus, as compared to control conditions, culturing on LM-421 improved β-cell function.

To conclude, our analysis indicates that β-cells respond differently to the various laminin isoforms naturally produced by pancreatic pericytes, and each of these BM components likely plays a different role in supporting islet structure and function. We further showed that LM-421, predominantly produced by pancreatic pericytes, induces β-cell gene expression and function in cultured islet cells.

## Discussion

ECM components, and more specifically BM components, are long known for their ability to support β-cell physiology. However, the source of these components was yet to be fully characterized. Here, we show that alongside ECs, pericytes are a significant source of the pancreatic IM and BM, including major constituents of the peri-islet membrane and the islet vascular BM. Specifically, we show that pericytes are the primary source of two of the three pancreatic laminin α chains, α2 and α4, and express these chains at significantly higher levels than pancreatic endothelial, endocrine, and exocrine cells do. Laminins produced by pericytes differentially stimulated the clustering of dispersed mouse islet cells. We found that the pericytic-produced laminin LM-421 induces the ex vivo expression of β-cell genes, including insulin, and improved the function of dispersed, cultured β-cells. Thus, our study points to the production of BM components as a potential mechanism through which pericytes regulate β-cells.

Pericytes and ECs cooperatively contribute to the vascular BM of many tissues^24^. Our results point to a similar interaction between these two cell populations in the pancreas. Interestingly, while some BM components, as collagen IV and the α4 laminin chain, are produced by both cell types, albeit at different levels, other components are produced by a single cell population. ECs, but not pericytes, produce the laminin α5 chain^2^ (Fig. 1; Table 1). In contrast, the laminin α2 chain is specific to pericytes (Fig. 2; Table 1). Thus, the pancreatic and islet BM compositions depend on ECM production by both ECs and pericytes.

The laminin α4 chain is restricted to the islets while absent from the exocrine pancreas^8,17,18^. This distinct pattern suggests that cells that comprise the islet vasculature produce different laminin isoforms than cells that encompass the exocrine vasculature, pointing to the heterogeneity of the pancreatic vascular cell populations. A direct comparison of endocrine- and exocrine-associated pericytes will allow a better understanding of such potential heterogeneity.

In addition to laminins, our study revealed that pancreatic pericytes produce other IM and BM components that impact β-cell physiology. Pericytes express fibronectin and collagen IV (Fig. 1); both were shown to affect β-cell function directly^1,2,4^. HSPGs, and particularly HSPG2 that is expressed by pancreatic pericytes (Fig. 1), were suggested to affect insulin production and secretion as well as β-cell proliferation indirectly through mediating growth factors activity^1^. Furthermore, pericytes are capable of producing HA (Fig. 1), which was shown to form an essential barrier, protecting the islets from immune infiltration^30,34^. Loss of the peri-islet membrane, specifically the loss of HA and HSPGs that comprise this membrane, precedes insulitis, and islets recovery further depends on the restoration of this barrier^30^. Pericytes were recently shown to have the potential to convert to myofibroblasts, which is characterized by aberrant ECM production and tissue fibrosis^28^. Whether abnormalities in pericytic ECM production contribute to immune cell infiltration and tissue fibrosis during diabetes remains to be further explored.

The various laminin isoforms were shown to affect β-cells differently^3,4^. LM-111, -411, and -511 were shown to stimulate β-cell proliferation and survival^2,32^. Previously, we showed that neonatal pancreatic pericytes stimulate β-cell proliferation in a β1-integrin -dependent manner^23^. However, here we were unable to detect changes in the number of β-cells cultured on α4 laminins (Fig. 3). Our gene expression analysis is in line with our previous reported expression of the laminin α2 chain by neonatal mesenchymal cells, which primarily represent pericytes^23,25,35^. However, while we previously reported the induction of β-cells gene expression upon culturing on Merosin (i.e., a mixture of LM-211 and LM-221 extracted from human placenta)^35^, here we did not observe this effect when culturing on recombinant human α2 laminins (Fig. 3). The inconsistencies in the observed effects of laminin isoforms could arise from the different laminin sources (i.e., recombinant vs. purified), as impurities of the native laminins and the presence of additional proteins, or differences in post-transcriptional modifications. An additional difference may arise from the potentially different concentrations of the applied laminins, which may suggest a dose-dependent effect on β-cell proliferation and gene expression.

This study points to the production of ECM components as a potential mechanism through which pericytes regulate β-cell physiology and glucose regulation. A direct proof of these components' requirement for pericyte-mediated glucose regulation may come from in vivo studies. As ECM components are essential for proper pancreas development, as well as for the functioning of various tissues^7,11,12,15,36,37^, this manipulation should be restricted to the adult pancreatic pericytes. However, currently available mouse models display either embryonic or whole-body expression^19,29^, thus precluding this line of in vivo experiments.

Human and mouse islets differ in both their cellular organization and the layering of the BM. In human islets, the vascular BM is covered by the invagination of the peri-islet membrane to form a double layer BM^9^. Thus, human β-cells are not directly contacting vascular BM components as α4 laminins^8^. Therefore, the response of human and mouse β-cells to specific vascular and peri-islet BM components in a physiological setting could significantly differ from their response in the dish. Nevertheless, generating and maintaining functional β-cells in vitro requires constructing a proper islet niche^38^. Thus, deciphering the composition and organization of the complex pancreatic and islet ECM network is fundamental for cell replacement therapy for diabetes.

## Material and methods

All methods were carried out in accordance with relevant guidelines and regulations.

The study was carried out in compliance with the ARRIVE guidelines.

### Mice

All experimental protocols were approved by the Tel Aviv University Institutional Animal Care and Use Committee (IACUC). *Nkx3.2*-Cre (Nkx3-2^tm1 (cre)Wez^) mice were a generous gift from Warren Zimmer (Texas A&M). *R26*-YFP (Gt(ROSA)26Sor^tm1 (EYFP)Cos^) mice were obtained from Jackson Laboratories. Wild-type mice were purchased from Envigo Ltd. (Jerusalem, Israel).

### Islet isolation

For islet isolation, Collagenase P (0.8 mg/ml; Roche) dissolved in RPMI (Gibco) was injected through the common bile duct into the pancreas of a euthanized mouse. Dissected pancreatic tissue was incubated for 11–15 min at 37 °C, followed by a gradient separation with Histopaque 1119 (Sigma) for 20 min at 1300 g. Islets were collected from the gradient interface, followed by their manual collection.

### Flow cytometry

Pancreatic cell isolation was performed as described^26^. Briefly, dissected pancreatic tissues were digested with 0.4 mg/ml collagenase P (Roche) and 0.1 ng/ml DNase (Sigma) diluted in HBSS for 30 min at 37ºC, followed by cells filtration. For cell sorting, pericytes were collected using FACS Aria (BD) based on their YFP expression in *Nkx3.2*-Cre;YFP mice. After immunostaining with PE-conjugated anti-PECAM1 antibody, ECs were similarly collected based on their fluorescent labeling. For cell counting and proliferation measurement, dispersed islet cells were fixed in 4% PFA for 15 min, immunostained with AlexaFlour 647-conjugated anti-insulin and FITC- conjugated anti-Ki67 antibodies, and analyzed using the volumetric counting feature of CytoFLEX flow cytometer (Beckman Coulter). For quantification of dead cells, unfixed cells were incubated with DAPI (Sigma) and analyzed using the volumetric counting feature of CytoFLEX flow cytometer (Beckman Coulter), when cells that uptake this dye were considered dead. Antibodies are listed in Supplementary Table 1.

### RNAseq analysis

We utilized a previously described RNA sequencing^21^ that was deposited in ArrayExpress (https://www.ebi.ac.uk/arrayexpress/experiments/E-MTAB-5325/). To characterize gene expression of pancreatic pericytes, cells were purified by flow cytometry from 10-week-old mice, as described above, and islets were isolated from age-matched non-transgenic mice. RNA was extracted using the PureLink RNA micro kit (Invitrogen) and subjected to RNA sequencing to obtain 1.5–2 × 10^7^ reads from each sample. RNA amplification, cDNA library preparation, deep RNA sequencing, and bioinformatics analysis were performed using commercial services (Otogenetics Co., Atlanta, GA). Raw reads (Fastq files) for each sample were aligned to the Bacteroides Fragilis using STAR v2.4.0 with default parameters, using the DNAnexus platform. After alignment, estimation of transcript abundance measures as FPKM values was performed using Cufflinks in the Tuxedo protocol. Gene expression FPKM values were then used for differential expression analysis using Cuffdif, a part of the Cufflinks package, to compare the different groups.

### Immunofluorescence

Dissected pancreatic tissues were fixed in 4% paraformaldehyde for 4 h. Tissue was transferred to 30% sucrose solution overnight at 4ºC, followed by embedding in Optimal Cutting Temperature compound (OCT, Tissue-Tek) and storage at -80ºC. Tissues were sectioned by a cryostat (Leica) into 11 μm thick section and stained with primary antibodies (Supplementary Table 1), followed by AlexaFluor secondary antibodies (Invitrogen). Images were acquired using an SP8 confocal microscope (Leica).

### qPCR analysis

RNA was extracted from isolated tissue and cells using PureLink RNA micro kit (Invitrogen) according to the manufacturer's protocol, followed by cDNA generation using a high capacity cDNA reverse transcription kit (Applied Biosystems). Gene expression levels were determined with Taqman and SYBR green assays (Applied Biosystems), using specific assays as listed in Supplementary Table 2, and normalized to GAPDH and Cyclophilin using a StepOne cycler (Applied Biosystems).

### Cell culture

Islets were isolated as described above. 50–70 Islets were dispersed to single-cell by incubation with 0.05% Trypsin and 0.02% EDTA solution (Biological Industries) at 37 °C for 5 min with agitation. Cells were culture for either 48 or 72 h in CMRL medium (Gibco) containing 10% FCS (Hyclone) and 1% Penicillin–Streptomycin solution (Biological Industries) on human recombinant laminins (BioLamina) or Poly-D-Lysine (PDL; Sigma) -coated 96-well plates (coating material is listed in Supplementary Table 3; plates coated for 2 h). Cells were collected using 0.05% Trypsin and 0.02% EDTA solution (Biological Industries) at 37 °C for 3 min.

### Morphometric analysis

Cells were imaged using Incucyte (Sartorius). Clusters size was measured by ImageJ software (NIH). Clusters smaller than a cell size were excluded from the analysis.

### Insulin secretion

Cells were collected, rinsed twice with RPMI, and pre-incubated for 30 min in RPMI 1640 (Gibco) supplemented with 0.1% BSA and 25 mM HEPES containing 1.67 mM glucose. Islets were then incubated in 1.67 mM glucose for 1 h, followed by additional incubation with 16.7 mM glucose for 1 h. After each incubation period, supernatant samples were collected, and secreted insulin levels were determined using mouse Ultrasensitive Insulin ELISA (Alpco, 80-INSMSU-E01). The ratio between insulin secreted in response to 1.67 and 16.7 mM glucose was determined per each well.

### Statistics

Paired data were evaluated using a two-tailed Student *t*-test.

## Supplementary Information

Supplementary Information.
