# Supplementary material for: Pericytes contribute to the islet basement membranes to promote beta-cell gene expression
Source: Sci Rep. 2021 Jan 27;11:2378. doi: 10.1038/s41598-021-81774-8 (PMC7840750; doi:10.1038/s41598-021-81774-8)
Supplement: Supplementary file 1 — Supplementary Information. [file 41598_2021_81774_MOESM1_ESM.pdf]

Supplementary data

Pericytes contribute to the islet basement membranes to promote beta-cell gene expression

Lina Sakhneny, Alona Epshtein, and Limor Landsman

Supplementary Table 1: List of primary antibodies

**Antigen**	**Host species**	**Manufacturer**	**Catalog number**	**Application**
Insulin	Guinea pig	Dako	IR00261-2	Immunofluorescence
Insulin, AlexaFluor647-conjugated	Rabbit	Cell Signaling Technology	9008S	Flow cytometry
Ki67, FITC-conjugated	Rat	eBioscience	11-5698-82	Flow cytometry
Laminin, α2 chain	Rat	Enzo Life Sciences	ALX-804-190-C100	Immunofluorescence
NG2	Rabbit	Millipore	AB5320	Immunofluorescence
PECAM1, PE-conjugated	Rat	BioLegend	102407	Flow cytometry

Supplemental Table 2: List of qPCR probes

Gene	Assay	Probe
Col4a1	Taqman	Invitrogen, Cat # Mm01210125_m1
Col4a2	Taqman	Invitrogen, Cat # Mm00802386_m1
Lama2	Taqman	Invitrogen, Cat # Mm00550083_m1
Lama4	Taqman	Invitrogen, Cat # Mm01193660_m1
Ins1	SYBR green	GGGTCGAGGTGGGCC,<br>CTGCTG GCCTCGCTTGC
Mafa	SYBR green	GCTGGTATCCAT GTCCGTGC,<br>TGTTTCAGTCGGATGACCTCC
GAPDH	Taqman	TGC ACC ACC AAC TGC TTAG,<br>GGA TGC AGG GAT GAT GTTC,<br>Probe: CA GAA GAC TGT GGA TGG CCC CTC
Cyclophilin	SYBR green	TGCCGCCAGTGCCATT,<br>TCACAGAATT ATTCCAGGATTC

Supplemental Table 3: List of plate coating components

**Protein**	**Manufacturer**	**Catalog**
Laminin-211	BioLamina	LN211-02
Laminin -21	BioLamina	LN221-02
Laminin-411	BioLamina	LN411-02
Laminin-421	BioLamina	LN421-02
Poly-D-Lysin	Sigma-Aldrich	P7405
